# Microwave-Assisted Synthesis of Zeolite A from Metakaolinite for CO_2_ Adsorption

**DOI:** 10.3390/ijms241814040

**Published:** 2023-09-13

**Authors:** Marilia R. Oliveira, Juan A. Cecilia, Daniel Ballesteros-Plata, Isabel Barroso-Martín, Pedro Núñez, Antonia Infantes-Molina, Enrique Rodríguez-Castellón

**Affiliations:** 1 Center for Studies in Colloidal Systems (NUESC), Laboratory of Materials Synthesis and Chromatography, Institute of Technology and Research (ITP), Tiradentes University (UNIT), Aracaju 49032-490, SE, Brazil; marilia.rafaele@souunit.com.br; 2 Department of Inorganic Chemistry, Crystallography and Mineralogy, Malaga University, 29071 Málaga, Spain; daniel.ballesteros@uma.es (D.B.-P.); ainfantes@uma.es (A.I.-M.); 3 Department of Chemistry, Institute of Materials and Nanotechnology, University of La Laguna, 38200 Tenerife, Spain; pnunez@ull.es

**Keywords:** kaolinite, zeolite type A, microwave synthesis, CO_2_ adsorption

## Abstract

The global demand for energy and industrial growth has generated an exponential use of fossil fuels in recent years. It is well known that carbon dioxide (CO_2_) is mainly produced, but not only from fuels, which has a negative impact on the environment, such as the increasing emission of greenhouse gases. Thus, thinking about reducing this problem, this study analyzes microwave irradiation as an alternative to conventional heating to optimize zeolite A synthesis conditions for CO_2_ capture. Synthesis reaction parameters such as different temperatures (60–150 °C) and different time durations (1–6 h) were evaluated. The CO_2_ adsorption capacity was evaluated by CO_2_ adsorption–desorption isotherms at 25 °C and atmospheric pressure. The results showed that the synthesis of zeolite A by microwave irradiation was successfully obtained from natural kaolinite (via metakaolinization), reducing both temperature and time. Adsorption isotherms show that the most promising adsorbent for CO_2_ capture is a zeolite synthesized at 100 °C for 4 h, which reached an adsorption capacity of 2.2 mmol/g.

## 1. Introduction

The current and future world scenario is one of the constant searches for sustainable technologies that can reduce the emission of CO_2_ in the atmosphere [1,2]. This is because CO_2_ is seen as the main reason for the increase in the generation of greenhouse gases (GHG), which leads to global warming and climate change [3,4]. The scientific community has reported that energy systems will be based on sustainable, low-carbon energy sources and free of polluting emissions [5]. These efforts are expected to try to mitigate environmental concerns in the future. For this reason, several industrialized and developing countries have been creating environmental regulations and achieving solutions to diminish CO_2_ emissions into the atmosphere [6,7,8]. Due to the simplicity, ease of use, and lower energy demand of the adsorption process, CO_2_ capture has been widely performed using solid adsorbents [9].

Regarding their physicochemical properties, high adsorption capacity, and favorable adsorption kinetics under mild operating conditions (0–100 °C, 0.1–1 bar CO_2_), zeolites are reference adsorbents for CO_2_ capture [10]. Zeolites are porous materials with a three-dimensional structure of crystalline aluminosilicate, which is obtained from the assembly of aluminate and silicate species. There are several sources for aluminum and silicon; however, it is necessary to search for and develop inexpensive aluminosilicate sources such as clay minerals. In this sense, kaolinite has been widely reported in the synthesis of zeolites, as it is a material that is easy to obtain and handle and presents promising results in the synthesis of crystalline zeolites and its application in adsorption processes [11,12]. Type A zeolite is one of the most common materials obtained from natural sources of Al and Si, as clay minerals, at low aging temperatures under hydrothermal conditions in a basic medium [13].

Seeking to optimize the synthesis process, microwave irradiation offers advantages over conventional heating, such as a highly uniform heating rate, a reduction in both reaction times and temperatures, and the ability to obtain pure products with good yields [14,15]. During microwave processing, microwave energy has the potential to uniformly heat large parts of the material, delivering energy exactly where it is needed, unlike conventional heating, where the tube walls are heated first [16]. According to Zeng et al., microwave synthesis will influence the crystalline structure of the material by controlling the reaction time and temperature [17]. The materials will possibly generate a kinetically favorable phase due to their fast crystallization rate.

Microwave synthesis of zeolite shows good results compared to conventional methodology. You et al. synthesized beta-type zeolites by the conventional hydrothermal method and by microwave irradiation [18]. The zeolite synthesized through microwave-assisted treatment was obtained after only 4 h (S_BET_ 463 m^2^/g and V_p_ 0.28 cm^3^/g), obtaining properties like the zeolite synthesized in 48 h by the hydrothermal method (S_BET_ 483 m^2^/g and V_p_ 0.29 cm^3^/g) [18]. In addition to showing a drastic reduction in synthesis time, this result was also attributed to the better hydrophilicity generated in this material [18]. Makgabutlane et al. synthesized zeolite A by microwave irradiation using coal ashes as precursors, varying the synthesis time, irradiation power, and Si/Al ratio [19]. The increase in microwave power and irradiation time favored the crystallization of phase A of the zeolite due to the sufficient energy needed to ensure the dissolution of Al and Si from the coal ashes and its subsequent assembly. A surface area of 29.54 m^2^/g and a cation exchange capacity of 3.10 m_eq_/g were achieved for zeolite A, suggesting a material with potential applications for adsorption or catalysis [19]. It is necessary to highlight that type A zeolite is highly microporous, and the adsorption values from N_2_ isotherms are relatively low due to the fact that this molecule is not well absorbed in narrow pores.

In addition, some studies also evaluate the combination of microwave and hydrothermal heating methods. Previous authors verified that with a combination of methods, it is possible to increase the crystallinity and surface area of zeolites, in addition to improving morphology and selectivity and obtaining smoother surfaces [20,21]. Notably, it is noticed that microwave-assisted methods present better results compared to conventional ones, providing a more ecological approach for the synthesis of zeolites in addition to reducing reaction times. In this study, the efficiency of microwave irradiation in the synthesis of type A zeolites based on kaolinite and its efficiency in the adsorption of CO_2_ are studied.

Thus, the motivation for using microwave irradiation in this study has mainly been the design of faster and more economically viable synthesis methods. In this context, the objective of this study is to evaluate the influence of microwave irradiation on the formation of zeolite by varying some synthesis parameters, such as time and temperature, and its performance in the adsorption of CO_2_.

## 2. Results and Discussion

### 2.1. Characterization

The synthetic conditions of the zeolitic materials obtained from kaolinite are compiled and labeled in Table 1.

**Figure 1:**
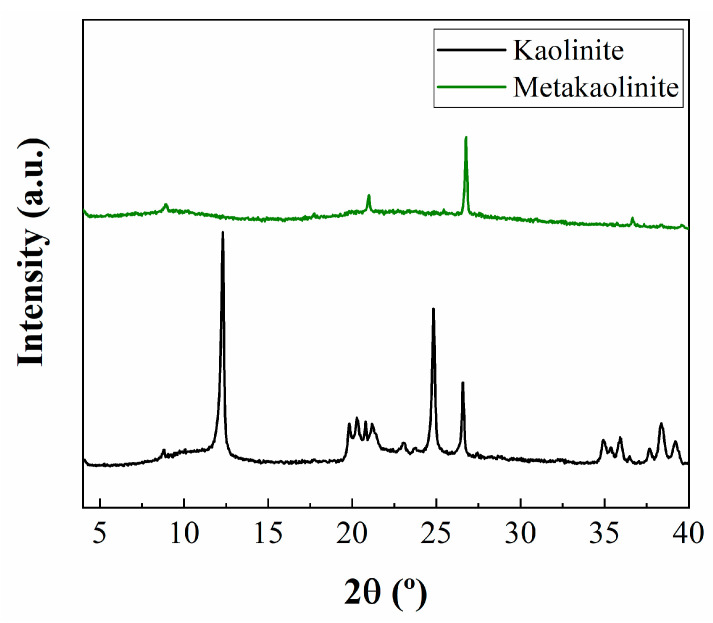
X-ray diffraction patterns of kaolinite and metakaolinite.

Figure 1 shows the XRD patterns of the kaolinite, metakaolinte, and synthesized materials. The raw material displays the typical diffraction peaks of kaolinite, with *d*_001_ diffraction located at 2θ of 12.3°, which implies a basal diffraction of 7.1 Å. In addition, the presence of a peak of 2θ at 26.6° confirms the existence of quartz as an impurity. In addition, several diffraction peaks located about 2θ ≈ 20° are also observed, which are ascribed to the presence of feldspars. The thermal treatment to form metakaolinite leads to an amorphous material due to the dehydroxilation of kaolinite and the collapse of the lamellar structure. However, the diffraction peaks ascribed to quartz remain after the thermal treatment.

**Figure 2:**
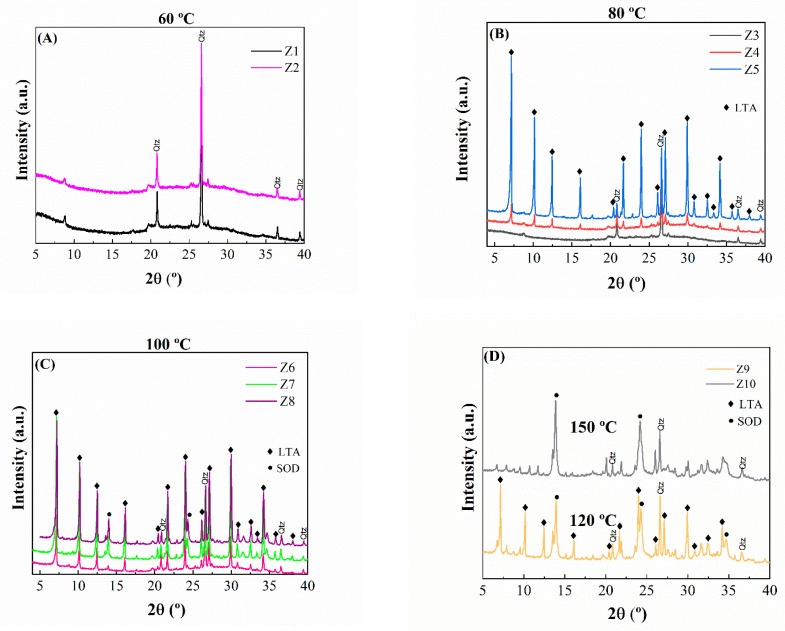
X-ray pattern of zeolites A synthesized from metakaolinite under microwave-assisted treatment under hydrothermal conversions at (**A**) 60 °C and 2 and 4 h, (**B**) 80 °C and 2, 4 and 6 h, (**C**) 100 °C and 2, 4 and 6 h and (**D**) 120 °C for 2 h and 150 °C for 1 h.

Regarding the zeolitic materials synthesized by microwave-assisted treatment, it is observed that it is not possible to observe any diffraction peaks ascribed to zeolitic materials at a temperature of 60 °C and a time of 2 or 4 h since the diffraction peaks detected are attributed to quartz, as was observed in kaolinite and metakaolinite (Figure 2A). From these data, it can be inferred that these conditions of temperature and time are not enough to obtain an aluminosilicate with high purity and crystallinity. Thus, the diffraction peaks observed at this temperature are ascribed to silica, which is not solubilized under microwave-assisted treatment under basic conditions. When the temperature is increased to 80 °C (Figure 2B), it is possible to obtain the characteristic peaks of the type A zeolite. The crystallinity increases according to the time of the microwave-assisted treatment, with the highest crystallinity at 6 h of treatment. Under these conditions, the diffraction peaks ascribed to quartz disappear due to the formation of silicate species, which are involved in the synthesis of zeolites. When the temperature is increased to 100 °C (Figure 2C), it is already possible to verify the characteristic pattern of the type A zeolite in a shorter reaction time (4 h). The treatment at a higher temperature (Figure 2D) causes a progressive loss of the typical diffraction peaks of type A zeolite, resulting in a new signal ascribed to another zeolite (sodalite). From these data, it can be inferred that the maximum temperature to obtain type A zeolite is 100 °C.

In general, with the increase in temperature and reaction time, the diffraction peaks of zeolite began to become more evident due to the formation of aluminate and silicate species, as well as its polymerization and assembly, which promoted the formation of zeolites with well-defined crystallinity. However, the assembly depends on the temperature since the XRD profiles show how type A zeolite is obtained until 100 °C since the use of higher temperatures promotes other assembling of the aluminate and silicate species under hydrothermal and basic conditions, forming sodalite or gismondine in minor proportions [22,23]. In any case, it should be highlighted that there is a notable reduction in the time of crystallization with the use of the microwave-assisted acid treatment in comparison to the conventional method, where 2–3 days are required at least. According to the data reported in Figure 2, it can be noted that the zeolite with the highest crystallinity is Z5.

In the study reported by You et al., these authors compared the synthesis of beta-type zeolites by the conventional hydrothermal method and by microwave irradiation [18]. The beta zeolite synthesized in a microwave reached high purity and crystallinity after only 4 h of treatment, while the zeolite synthesized by the hydrothermal method requires conventional heating for 48 h. In the same way, Xia et al. synthesized EMT-zeolite by microwave and conventional hydrothermal heating, indicating that microwave heating drastically reduced the synthesis time from 72 h to only 30 min, achieving agglomerated samples with a high crystallinity in short times [20]. A comparison of microwave treatment with conventional hydrothermal heating revealed that in conventional hydrothermal heating, these authors would need heating for at least 72 h to obtain a zeolite with better crystallinity. In this work, it was already possible to verify the efficiency of microwaves in this process, where the synthesis time was drastically reduced. This decrease in synthesis time is related to localized superheating generated by microwave irradiation, leading to faster dissolution of the aluminosilicate into aluminate and silicate species and their subsequent assembly to form well-defined zeolites.

**Figure 3:**
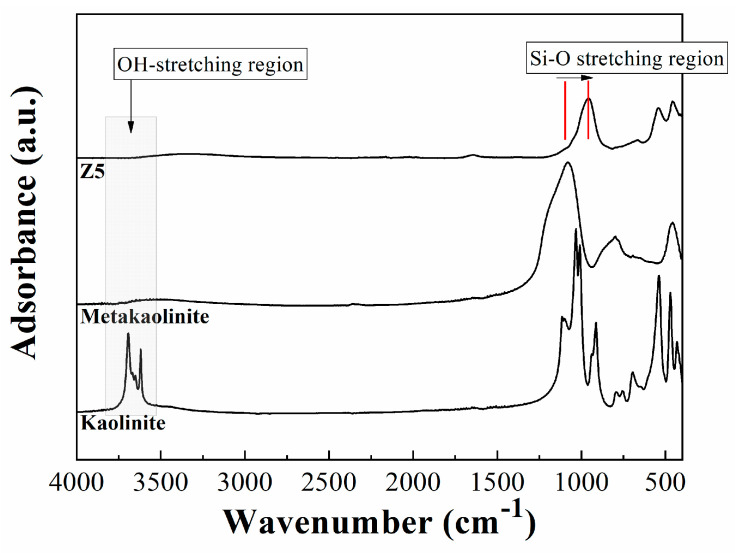
ATR spectra of kaolinite, metakaolinite, and zeolite synthesized at 80 °C for 6 h (Z5).

The FTIR spectra of zeolites A, along with kaolinite and metakaolinite, are shown in Figure 3. The analysis of the OH-stretching region between 3800 and 3500 cm^−1^ shows four defined bands. The band located about 3690 cm^−1^ is attributed to an in-phase symmetric stretching vibration mode; the smaller bands located at 3670 and 3650 cm^−1^ are ascribed to out-of-plane stretching vibration modes; while the band located about 3618 cm^−1^ is assigned to the inner hydroxyl groups [24,25]. The study of the kaolinite spectrum between 1750 and 450 cm^−1^ shows a set of well-defined bands between 1130 and 990 cm^−1^, which are ascribed to Si-O stretching vibration modes [24]. In the same way, other bands with a maximum close to 940 and 910 cm^−1^ are also observed, which are attributed to the existence of Al_2_OH bending modes of inner and surface –OH groups [24]. The band centered at about 540 cm^−1^ is attributed to Si-O-Al bending vibration mode, while the band located at about 470 cm^−1^ is assigned to Si-O-Si bending vibration mode [24].

The thermal treatment of the kaolinite to form metakaolinite provokes the dehydroxilation of the –OH groups in kaolinite, causing a collapse in its structure. Thus, the typical bands of the –OH stretching modes disappear [26]. In the same way, it is also noteworthy the loss of some bending vibration modes due to the absence of –OH groups [25].

As for zeolites A, it is verified that in the region of 460, 550, and 1000 cm^−1^, the vibration bands are attributed to Si-O, Si-O-Al, and Si-O-Si elongation. The band located at 1650 cm^−1^ is associated with interstitial water bending, and the broad band located between 3000 and 3500 cm^−1^ is attributed to intra- and intermolecular hydrogen bonds [25,27]. The FTIR spectrum corroborates the results obtained by the XRD analysis, confirming the synthesis of zeolitic material.

**Figure 4:**
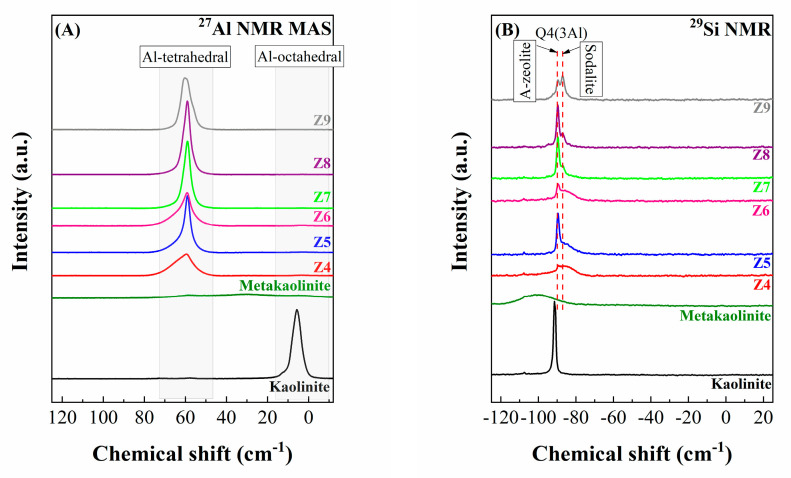
(**A**) ^27^Al MAS NMR and (**B**) ^29^Si NMR spectra of kaolinite, metakaolinite, and zeolites synthesized at different temperatures and reactional times in a microwave reactor.

Both kaolinite and metakaolinite, as well as the zeolites synthesized under hydrothermal conditions, were also analyzed by ^27^Al and ^29^Si MAS NMR (Figure 4).

The ^27^Al MAS NMR spectrum (Figure 4A) of the raw kaolinite shows a peak of about 6 ppm, which is ascribed to an octahedral environment, which agrees with the structure of phyllosilicates where Al-species are in the octahedral sheet of the clays. After the thermal treatment of the kaolinite to promote its dehydroxylation, ^27^Al MAS NMR of metakaolinite does not show any defined bands, confirming the collapse of the layered structure and the formation of an amorphous aluminosilicate after the thermal treatment. When metakaolinite is subjected to a microwave-assisted treatment under hydrothermal and basic conditions, the ^27^Al MAS NMR spectra show a peak centered about 59 ppm, probing the presence of tetrahedrally coordinated aluminum bonded by oxygen bridges to four Si atoms [28,29]. In many cases, a shoulder at a higher chemical shift is observed. This change is attributed to the splitting of the peaks when subjected to a high field [30].

Regarding the study of the samples by ^29^Si MAS NMR (Figure 4B), the spectrum of the kaolinite sample shows a well-defined signal located at −91 ppm. This value agrees with the literature, where Si-species display a tetrahedral coordination where three are bonded to oxygen atoms and the last one is non-bonded to oxygen [31]. The thermal treatment to form metakaolinite causes loss-defined signals in its ^29^Si MAS NMR spectrum, obtaining a broad band between −120 and −80 ppm, confirming the heterogeneity of the Si species [32]. After the microwave-assisted treatment to obtain the zeolites, a band located at −89.4 ppm is observed, which is ascribed to the Q4(3Al) environment. In addition, it is also noteworthy how an increase in the temperature in the treatment caused a change in the assembly of the aluminosilicate from type A zeolite to sodalite. This supposes a slight shift of the Q4(3Al) environment from −89.4 ppm to −87.3 ppm [18,25]. It is also verified that at higher temperatures or higher reaction times, the peak of Al and Si becomes more intense, which is related to the conditions of synthesis and formation of the zeolite, also confirming that in extreme conditions of time and temperature, it is not possible to obtain the chemical composition of type A zeolites.

**Figure 5:**
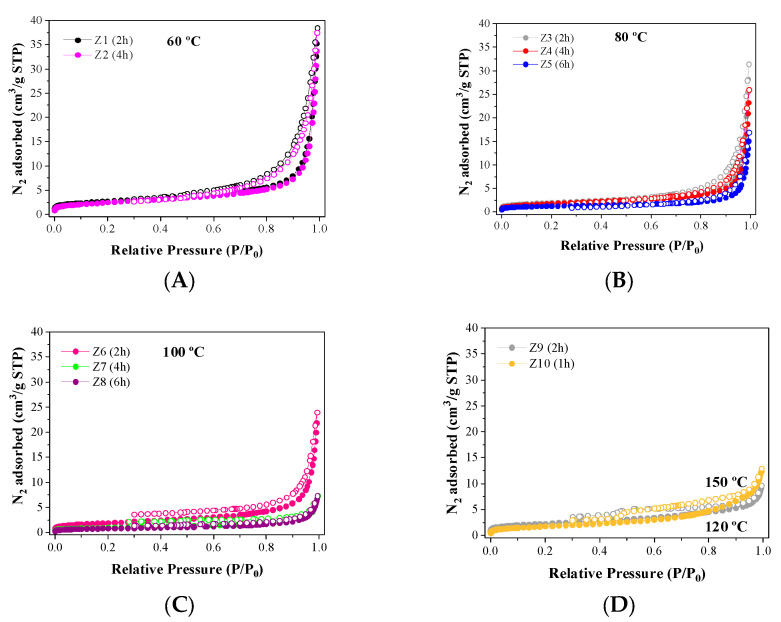
N_2_ adsorption (full dots) and desorption (hollow dots) isotherms (−196 °C) of the zeolites synthesized at (**A**) 60 °C, (**B**) 80 °C, (**C**) 100 °C and (**D**) 120 and 150 °C.

The analysis of the textural properties was carried out by N_2_ adsorption–desorption at −196 °C and CO_2_ adsorption isotherms at 0 °C (Figure 5). By analyzing the nitrogen adsorption isotherms, it becomes evident that most zeolites exhibit a type I isotherm, classifying them as predominantly microporous materials [29]. However, in the case of zeolites synthesized for 2 or 4 h, their adsorption–desorption curves take on the characteristic traits of type IV isotherms. These isotherms feature a hysteresis loop, suggesting the appearance of mesoporous properties. This observation may be attributed to the short synthesis duration under these conditions, which seemingly hindered the formation of microporous zeolites, as corroborated by the XRD results (Figure 2).

In Table 2, it is noted that the amount of N_2_ adsorbed is very low in kaolinite, metakaolinite, and zeolitic materials. In the case of kaolinite, the packing of the tetrahedral-octahedral (TO) sheets hinders access to the CO2 molecules due to its structure. These poor values are also observed when metakaolinite is formed. Regarding the zeolitic materials, the low values must be ascribed to the high microporosity of their frameworks in such a way that N_2_ molecules cannot access and reach the equilibrium conditions to elaborate an adsorption isotherm. These data corroborate the S_BET_ and pore volume values reported in Table 2 since the surface area of the obtained zeolites varied from 2.8 to 9.7 m^2^/g, the pore volume (V_p_) ranged between 0.005 and 0.019 cm^3^/g, and the maximum micropore volume (V_mp_) is only 0.0010 cm^3^/g, so the mesoporosity of the zeolitic materials can be considered insignificant. These poor values agree with the data reported by XRD (Figure 2) since the diffractograms reveal the formation of type-A or sodalite zeolites after microwave-assisted treatment, which are of high microporosity.

**Figure 6:**
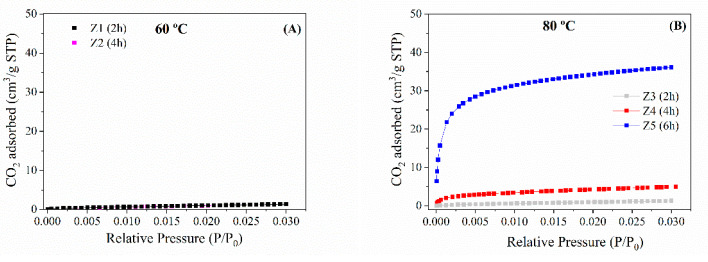

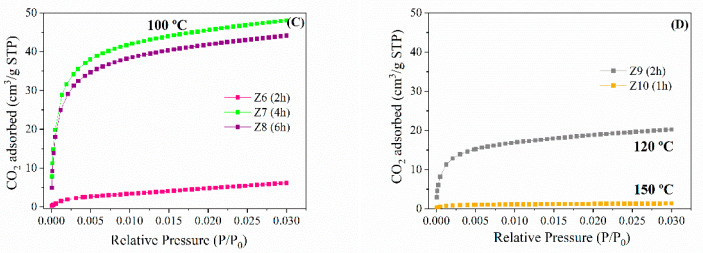
CO_2_ adsorption and desorption isotherm (measured at 0 °C) of the zeolites synthesized at (**A**) 60 °C, (**B**) 80 °C, (**C**) 100 °C and (**D**) 120 and 150 °C.

Considering the poor values obtained from N_2_ adsorption–desorption isotherms at −196 °C, an alternative to determining the microporosity of highly microporous materials is the analysis of the CO_2_ isotherms at 0 °C (Figure 6), since CO_2_ molecules can more easily access the micropores due to their high quadrupole moment (−14.27·10^−40^ C m^2^) [33]. However, N_2_ molecules have a lower quadrupole moment (−4.65·10^−40^ C m^2^) [33], which makes it difficult to reach equilibrium conditions at low relative pressures, that is, for those microporous materials. Other authors have also pointed out that N_2_ and CO_2_ have similar critical dimensions. However, CO_2_ adsorption is carried out at a temperature higher than its boiling point, and the gas molecules can enter the narrowest porosity of the solid [34].

The textural properties, determined by CO_2_ adsorption isotherms at 0 °C (Table 3), show that those zeolites with higher crystallinity (Figure 2) also display higher micropore volumes. Thus, sample Z7, which presents an A-zeolite structure with the highest crystallinity, also reaches the largest microporosity values with a micropore volume of 0.1177 cm^3^/g and an equivalent surface area of 294 m^2^/g. In general, the analysis of the microporosity follows the same trend as that observed from the XRD data since the crystallinity and the microporosity increase when the temperature increases due to the formation of well-defined type-A zeolite. The best values and ordered structures were obtained at 100 °C. The use of higher temperatures in the microwave-assisted treatment leads to the formation of other zeolites with poorer crystallinity and wider pore sizes in their cages. This implies that those samples synthesized at 120 and 150 °C display poorer textural properties.

**Figure 7:**
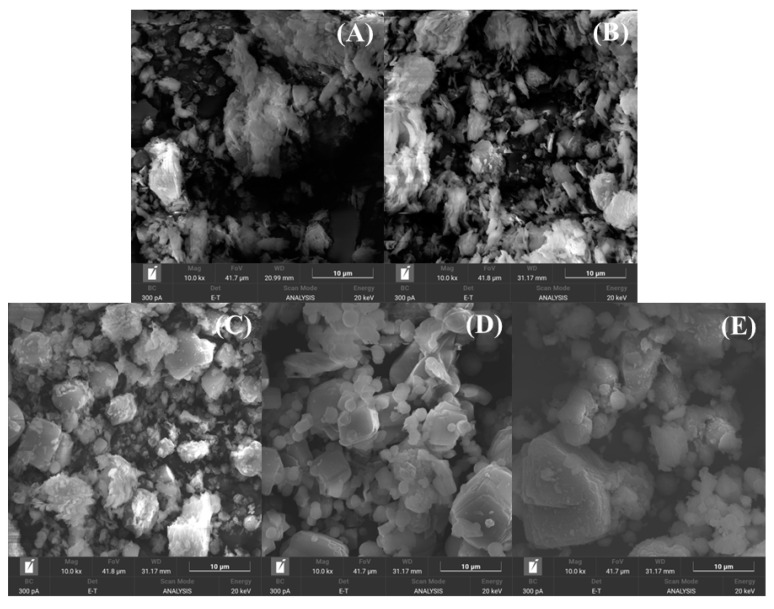
SEM images of kaolinite (**A**), metakaolinite (**B**), Z6 (**C**), Z7 (**D**), and Z8 (**E**) samples.

Considering that the materials with higher microporosity are those synthesized at 100 °C, the morphologies of these samples were studied by SEM (Z6, Z7, and Z8). The morphologies of these aluminosilicates were compared with the starting kaolinite and metakaolinite (Figure 7). Figure 7A shows how the kaolinite sheets are stacked, although it should be noted that clay minerals are generally disordered because they are materials that have been subjected to environmental effects. This stacking decreases for the metakaolinite sample due to dehydroxylation of the –OH groups (Figure 7B), causing further structural disorder. In the case of zeolites synthesized by microwave-assisted treatment (Figure 7C–E), the structure of the materials differs in comparison to the starting materials, obtaining materials with globular or cubic structures. In fact, sample Z7 is the material that presents the highest crystallinity and microporosity, and in the same way, this sample presents a more defined morphology, although structures with two dimensions are observed (Figure 7D).

### 2.2. CO_2_ Adsorption Studies

**Figure 8:**
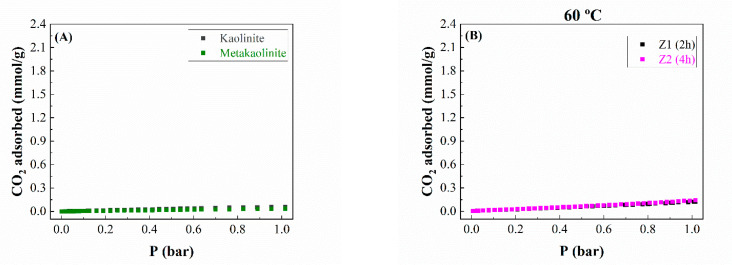

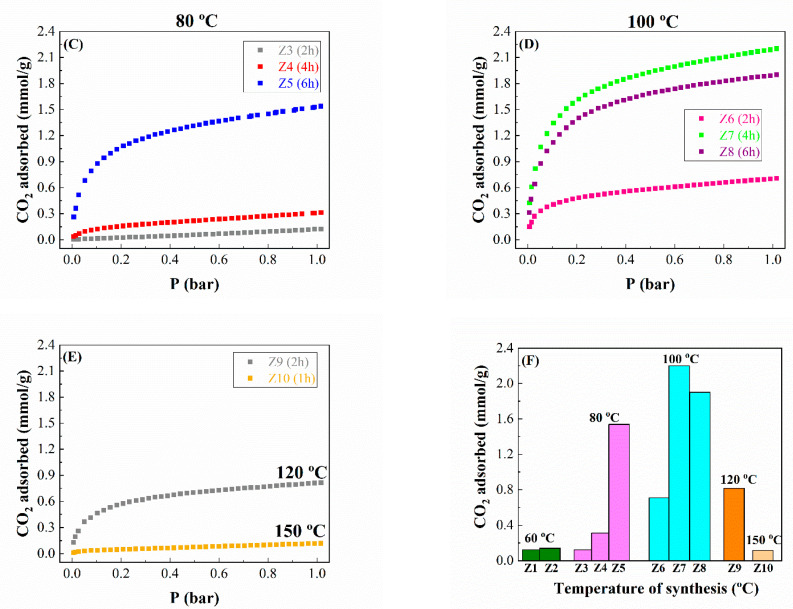
CO_2_ adsorption/desorption isotherms at 25 °C for (**A**) commercial zeolites and the zeolites synthesized at (**B**) 60 °C, (**C**) 80 °C, (**D**) 100 °C, (**E**) 120 and 150 °C. (**F**) Comparison of CO_2_ uptake for all prepared zeolites.

Once the materials have been characterized, the CO_2_ adsorption capacity of these zeolitic materials has been evaluated in volumetric equipment at 25 °C between 0 and 1 bar of pressure (Figure 8).

Firstly, the adsorption capacity of the starting materials, i.e., kaolinite and metakaolinite, was evaluated (Figure 8A). In both cases, the CO_2_ adsorption capacity is very poor, reaching values of 0.06 and 0.04 mmol/g at 25 °C and 1 bar of pressure for kaolinite and metakaolinite, respectively. These data agree with the literature since kaolinite hardly adsorbs CO_2_ molecules in its interlayer spacing and surface, while metakaolinite displays an amorphous structure without porosity where CO_2_ molecules are not adsorbed in the voids between particles, as was observed by XRD (Figure 1) and its textural properties (Table 3) [35].

When metakaolinite is subjected to microwave-assisted treatment under hydrothermal conditions at 60 °C (Figure 8B), the obtained materials hardly display crystallinity as detected by XRD (Figure 2) and poor textural properties (Table 3). This implies that the CO_2_ adsorption capacity values of the materials synthesized at 60 °C hardly differ from those obtained for the raw kaolinite and metakaolinite (Figure 8A). Thus, the CO_2_ adsorption capacity for Z1 and Z2 samples is in the range of 0.11–0.13 mmol/g at 1 bar of pressure and 25 °C.

The use of a higher temperature in the microwave-assisted treatment (80 °C) seems to improve the CO_2_ adsorption capacity (Figure 8C). In fact, a longer microwave-assisted treatment time produces a notable improvement in the CO_2_ adsorption capacity due to the formation of a more crystalline type A zeolite whose microporous structure is better defined. Thereby, the maximum CO_2_ adsorption capacity has been obtained by sample Z6 after 6 h of microwave-assisted treatment, reaching a value of 1.53 mmol/g at a pressure of 1 bar and a temperature of 25 °C. In this sense, it should be noted that the profile of the adsorption isotherms also differs since the isotherms where the adsorption capacity is poorer are practically linear; however, the most microporous materials adsorb a greater amount of CO_2_ at lower pressures. This implies that the isotherm loses its linearity due to stronger adsorption between CO_2_ and the adsorbate.

This trend is more pronounced when microwave-assisted heating is carried out at 100 °C (Figure 8D). In this case, the maximum CO_2_ adsorption capacity is obtained after 4 h of treatment, achieving a value of 2.18 mmol/g at 1 bar of pressure and 25 °C. The use of a longer reaction time does not improve the adsorption capacity due to the formation of a material with poorer textural properties.

The use of higher treatment temperatures considerably worsens the adsorption capacity (Figure 8E) because other zeolites are formed whose structures are less crystalline or whose pores are more open; these materials adsorb less CO_2_.

From these data, it can be concluded that the optimization of the temperature and time of the microwave-assisted treatment plays a key role in the synthesis of the type of zeolite as well as its crystallinity, as was suggested by X-ray diffraction (Figure 2). The CO_2_ adsorption data also reveal that it is possible to reach CO_2_ adsorption capacities similar to and, in some cases, even superior to those of zeolites synthesized by the conventional method [18,25].

In previous studies, You et al. evaluated the CO_2_ adsorption capacity of beta zeolite synthesized by microwave-assisted treatment and by the conventional hydrothermal method and found the efficiency of microwave irradiation [18]. The CO_2_ adsorption tests were investigated at a temperature of 40 °C and an absolute pressure of 1. Beta-zeolite synthesized by microwave-assisted treatment was quickly synthesized in 4 h (S_BET_: 463 m^2^/g and V_P_: 0.28 cm^3^/g), obtaining properties like beta-zeolite obtained through the conventional method after 48 h (S_BET_: 483 m^2^/g and V_P_: 0.29 cm^3^/g). However, the beta-zeolite synthesized by microwave-assisted treatment leads to an adsorption capacity of 2.16 mmol/g and a selectivity in CO_2_ of 17.1%, better than the beta-zeolite obtained by the conventional method (1.94 mmol/g and 14.5%). It should be noted that the textural results were similar in both cases, although the microwave-assisted treatment diminishes the synthesis time and increases the CO_2_ adsorption capacity in comparison to the hydrothermal method.

These results from this study confirm the advantage of microwave-assisted furnaces for the synthesis and subsequent application in adsorption processes, which, in addition to leading to promising results, considerably reduce the synthesis time for the obtained product. In this sense, Cecilia et al. reported how the time and temperature of synthesis can be key parameters in the synthesis of zeolites from metakaolinite and the CO_2_ adsorption capacity [25]. These authors synthesized type A zeolites under different conditions at times of 6, 20, 48, 96, and 168 h and at temperatures of 60, 80, 100, and 120 °C by the conventional hydrothermal method. It should be noted that increasing the synthesis time improved the adsorption capacity (q_ads_), reaching the highest adsorption capacity for zeolites synthesized at 48 and 96 h with q_ads_ of 2.48 and 2.53 mmol/g at 25 °C and 1 bar of pressure. Analyzing the influence of temperature, this study verified that below 80 °C and above 100 °C, the CO_2_ adsorption capacity was smaller. Thus, the zeolites synthesized at 60 °C reached a q_ads_ = 0.80 mmol/g, while those synthesized at 120 °C achieved a q_ads_ = 0.75 mmol/g. The comparison of these values with those obtained by the microwave-assisted acid treatment reveals that the analysis of the CO_2_ adsorption capacity at low temperatures (60 °C) is higher in the case of the conventional method, while in the study at higher temperatures (120 °C), both methodologies display similar adsorption capacities. When the samples are synthesized at the optimum temperature (100 °C), the highest CO_2_ adsorption capacity is 2.48 mmol/g at a pressure of 1 bar and 25 °C, although it takes 96 h of the conventional hydrothermal method to obtain a zeolite with high crystallinity to reach this adsorption capacity. With the microwave-assisted treatment, it takes only 4 h to reach an adsorption capacity of 2.18 mmol/g under similar adsorption conditions. Thus, it can be concluded that the results obtained in this work display advantages over syntheses that use adsorbent zeolite prepared by conventional methodologies without the use of microwave irradiation.

**Figure 9:**
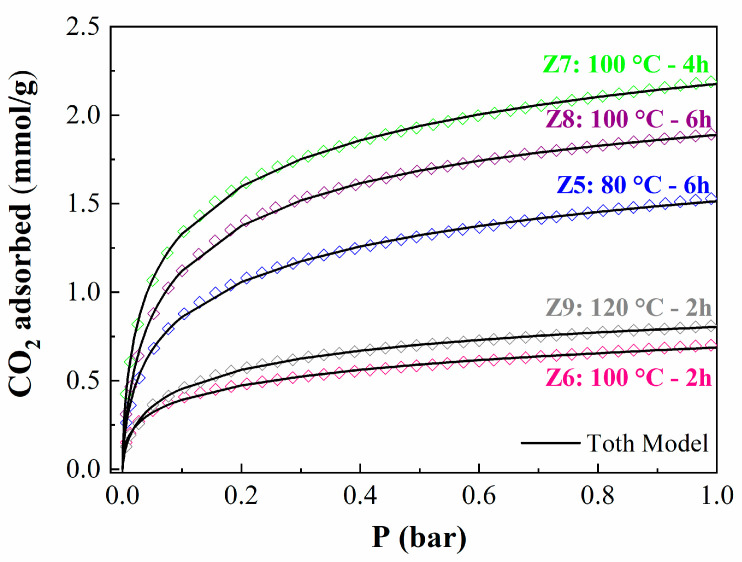
CO_2_ adsorption isotherms of zeolites at 25 °C and 1 bar, fitted to the Toth model.

Finally, the CO_2_ adsorption isotherms at 25 °C fitted well to the Toth model (Figure 9). The Toth model assumes that adsorption occurs exclusively with sub-monolayer coverage [25] and shows good agreement with the experimental data, being able to accurately predict the sites of CO_2_ adsorption.

Table 4 presents the values of the maximum CO_2_ adsorption capacity (q_m_), the affinity between adsorbate and adsorbent (b), and the degree of homogeneity of the adsorbents (n). Note that zeolites exhibit heat of adsorption between ΔH = −32.0 and −39.8 kJ/mol. These values are in the same range as those observed for the zeolites synthesized by the conventional method [36]. Regarding heterogeneity, the parameter n is far from unity, varying between 0.35 and 0.48. This implies that there are prone sites for the adsorption of CO_2_, probably the narrow pores of the zeolite, while the larger pores should barely retain CO_2_, in such a way that the adsorbents are quite heterogeneous, as observed in the SEM images (Figure 7). Another important parameter to be analyzed is the b parameter, which is related to the energetic interaction between the adsorbent and the adsorbate [37]. The b values show how the b parameter is directly related to the adsorption capacity. In this sense, the data reported in Table 4 suggest that those materials with higher crystallinity and narrow pore diameters cause a strong interaction between the adsorbate (type-A zeolite) and the CO_2_ molecules, implying a higher CO_2_ adsorption capacity. Being the sample with a chemical formula of Na_12_Si_12_Al_12_O_48_ 27H_2_O, it has the best CO_2_ adsorption capacity.

## 3. Materials and Methods

### 3.1. Materials

Kaolinite (a silicon- and aluminum-rich clay mineral) was obtained from Vimianzo deposits (Galicia, Spain). Sodium hydroxide (NaOH, ACS reagent ≥ 97.0%, VWR, Radnor, PA, USA). The gases employed in the CO_2_ adsorption studies were CO_2_ (99.999%, Air Liquide, Spain), N_2_ (99.9999%, Air Liquide, Spain), and He (99.999%, Air Liquide, Spain).

### 3.2. Synthesis of Zeolites

The kaolinite was previously calcined at 600 °C for 4 h in a furnace to convert it into metakaolinite. This temperature was selected in accordance with previous studies reported in the literature for various kaolinites [38,39,40]. Then, the metakaolinite was added to a 3.0 M NaOH aqueous solution and stirred for 15 min at room temperature for homogenization. The solid–liquid ratio used in this process was 2 g of metakaolinite per 50 mL of alkaline solution. The stirred mixture was placed in a Teflon container and inserted into the microwave reactor (ETHOS, Milestone, Denmark), varying the temperatures (60 °C to 150 °C) and times (0.5 to 6 h) of synthesis, as detailed in Table 1. After microwave-assisted treatment, the samples were subjected to centrifugation at 1500 rpm for 25 min. Subsequently, the solids were carefully washed several times with deionized water (approximately 300 mL) to remove any remaining moisture and alkaline residue. Finally, the samples were dried in an oven overnight at 60 °C [13].

### 3.3. Characterization

X-ray powder diffraction patterns (XRD) were collected on a PANanalytical EMPYREAN automated diffractometer. Powder patterns were recorded in θ-θ transmission configuration by emplacing the sample between two kapton foils and using a focusing mirror and the PIXcel 3D detector (working in 1D mode) with a step size of 0.013° (2θ). The powder patterns were recorded between 0.5 and 10 degrees in 2θ, with a total measuring time of 60 min.

The Fourier transform infrared (FTIR) spectra were collected in a Vertex70 (Bruker, Germany) equipped with a Golden Gate Single Reflection Diamond ATR System accessory. For the acquisition of the spectra, a standard spectral resolution of 4 cm^−1^ was used in the spectral range of 4000–500 cm^−1^, as well as 64 accumulations.

^29^Si MAS-NMR and ^27^Al MAS NMR were recorded at RT on an AVANCEIII HD 600 (Bruker AXS) spectrometer using a double resonance DVT probe of 4.0 mm at a spinning rate of 13 kHz. The ^27^Al MAS NMR spectra were also performed with proton decoupling (continuous wave sequence) by applying a single pulse (π/12), an excitation pulse of 1 μs, and a 5 s relaxation delay to obtain 200 scans. ^29^Si MAS NMR spectra were recorded with an 8 ms 90° pulse and a 60 s delay with 1H decoupling (^29^Si high-power decoupling dec with cw decoupling sequence for Si) and summing up 1000 scans.

Nitrogen adsorption/desorption measurements were performed at liquid N_2_ temperature (−196 °C) with an ASAP 2420 apparatus (Micromeritics, Norcross, GA, USA). In a first study, the specific surface area was determined by the Brunauer–Emmett–Teller equation (BET) from the N_2_-adsorption isotherms at −196 °C in the range of relative pressures of 0 to 1 [41]. The pore volume and pore size distribution were calculated using desorption branches of nitrogen isotherms by Barret–Joyner–Halenda (BJH). The total pore volume was calculated from adsorbed N_2_ at a relative pressure (P/P_0_) = 0.99 [42].

The microporosity of the samples was determined from their CO_2_ adsorption isotherms at 0 °C using a Micromeritics 2420 apparatus. Prior to the studies to determine the microporosity, the solids were degassed at 150 °C overnight. After the adsorption isotherms, both the micropore volume and its equivalent surface area were determined using the Dubinin–Astakhov equation [43].

### 3.4. CO_2_ Adsorption

The adsorption tests were used to evaluate the CO_2_ capture capacity of the zeolites. Before the measurements, samples were outgassed at 110 °C and 10^−4^ mbar. Adsorption/desorption isotherms were measured with a Micromeritics ASAP 2020 Analyzer (i.e., volumetrically) at 25 °C, all under absolute pressure ranging to 1 bar. The purity of the CO_2_ used in the tests was 99.998%.

### 3.5. Adsorption Model

The Toth model allows a good description of many systems with sub-monolayer coverage [44]. Toth’s isothermal equation is

$$q_{i}=q_{m,i}\frac{b_{i}*P}{{\left[1+{\left(b_{i}*P\right)}^{n_{i}}\right]}^{{\frac{1}{n}}_{i}}}$$

where $q_{i}$ and $q_{m,i}$ are the amount adsorbed and the maximum adsorption capacity of component *i*; $b_{i}$ and $n_{i}$ are specific parameters for adsorbate–adsorbent pairs; and *P* is the pressure. This isotherm assumes that the adsorption occurs in only one layer and allows the interaction between the adsorbed molecules.

## 4. Conclusions

The utilization of microwave irradiation in the synthesis of zeolite A from metakaolinite has shown its potential to achieve faster and more cost-effective methods. The outcomes of this study show the advantages of microwave-assisted synthesis, not only in expediting the production of zeolites but also in enhancing their adsorption capabilities for CO_2_. This approach yielded zeolites of high crystallinity and purity and revealed a significant correlation between synthesis time and temperature within the microwave reactor.

Through the CO_2_ adsorption isotherms, it was possible to analyze that the adsorption capacity of zeolites increases with increasing temperature, except for temperatures higher than 120 °C due to the formation of mixtures of phases whose pore diameter is larger. The zeolite A synthesized under microwave irradiation at 100 °C for 4 h exhibited the highest CO_2_ adsorption capacity (q_ads_ = 2.18 mmol/g at 25 °C and 1 bar pressure). This result is particularly noteworthy as it substantially outperforms the results achieved in many prior studies that demanded significantly longer synthesis times, ranging from 24 to 96 h.

Thus, it was seen that microwave irradiation not only accelerates the synthesis of zeolites but also obtains adsorbents with a greater number of active sites available for CO_2_ adsorption and, consequently, a greater adsorption capacity than zeolites synthesized by the conventional method.

## Figures and Tables

**Table 1:** Synthesis conditions of zeolite A used in the microwave-assisted hydrothermal reactor. (All samples display similar chemical composition; only temperature and time differ.)

Samples	Time (h)	Temperature (°C)
Zeolite A 60 °C–2 h: Z1	2	60
Zeolite A 60 °C–4 h: Z2	4	
Zeolite A 80 °C–2 h: Z3	2	80
Zeolite A 80 °C–4 h: Z4	4	
Zeolite A 80 °C–6 h: Z5	6	
Zeolite A 100 °C–2 h: Z6	2	100
Zeolite A 100 °C–4 h: Z7	4	
Zeolite A 100 °C–6 h: Z8	6	
Zeolite A 120 °C–2 h: Z9	2	120
Zeolite A 150 °C–1 h: Z10	1	150

**Table 2:** Textural properties determined from N_2_ adsorption isotherms at −196 °C.

Samples	S_BET_ (m^2^/g)	S_mp_ (m^2^/g)	V_P_ (cm^3^/g)	V_MP_ (cm^3^/g)
Kaolinite	10.8	1.40	0.022	0.0011
Metakaolinite	12.2	1.70	0.024	0.0012
Z1	9.7	2.13	0.019	0.0010
Z2	8.9	1.35	0.017	0.0005
Z3	6.9	1.58	0.013	0.0007
Z4	6.4	0.88	0.011	0.0003
Z5	4.5	1.78	0.007	0.0007
Z6	6.8	0.7	0.013	0.0003
Z7	3.4	0.78	0.005	0.0003
Z8	2.8	0.89	0.005	0.0004
Z9	8.1	2.14	0.009	0.0010
Z10	6.4	--	0.012	--

**Table 3:** Textural properties determined from CO_2_ adsorption isotherms at 0 °C.

Samples	V_MP_ (cm^3^/g)	Equivalent Surface Area (m^2^/g)
Kaolinite	0.0008	2
Metakaolinite	0.0003	1
Z1	0.0029	7
Z2	0.0029	7
Z3	0.0027	7
Z4	0.0104	26
Z5	0.0867	216
Z6	0.0140	35
Z7	0.1177	294
Z8	0.0967	241
Z9	0.0490	122
Z10	0.0028	7

**Table 4:** Maximum CO_2_ storage capacity (q_m_), adsorbate–adsorbent affinity (b), and homogeneity grade (n) for all analyzed samples obtained as Toth equation fitting parameters.

Samples	qm (mmol/g)	B (bar^−1^)	n	(ΔH) (kJ/mol)
Z5	2.65	5.62 × 10^−5^	0.35	−35.2
Z6	2.42	5.48 × 10^−5^	0.38	−39.8
Z7	3.39	7.49 × 10^−5^	0.36	−36.1
Z8	2.60	5.59 × 10^−5^	0.48	−33.6
Z9	1.35	1.61 × 10^−4^	0.37	−32.0

## Data Availability

The data presented in this study are available on request from the corresponding author.
